# Cross-cultural adaptation and psychometric validation of the Flodén ATODAI instrument in the North American context

**DOI:** 10.1186/s12912-020-00444-8

**Published:** 2020-06-22

**Authors:** Anne Flodén, Maria Stadtler, Stephanie E. Jones Collazo, Tom Mone, Rick Ash, Bengt Fridlund

**Affiliations:** 1 Department of Anesthesiology & Department of Research, Sodra Alvsborg Hospital, Bramhultsvagen 53, 501 82 Boras, SE Sweden; 2 Institute of Health and Care Sciences, University of Gothenburg, Gothenburg, Sweden; 3 Association of Organ Procurement Organizations (AOPO), Vienna, USA; 4 Mount Saint Mary’s University, Los Angeles, CA USA; 5 OneLegacy, Los Angeles, CA USA; 6 Centre for Interprofessional Collaboration within Emergency care (CICE), Linnaeus University, Campus Växjö, Växjö, Sweden

**Keywords:** Instrument development, Translation and cross-cultural adaptation, Advocacy, Attitude, Organ donation, Nursing, Intensive care

## Abstract

**Background:**

Intensive and critical-care nurses are the key to successful donor management in the critical-care setting. No studies measuring attitudes toward organ donor advocacy existed before 2011, when the 51-item Swedish “Attitudes Toward Organ Donor Advocacy Scale” was developed. The aim of this study was to translate, adapt and establish the psychometric properties of the North American version of the Flodén ATODAI (Attitudes Toward Organ Donor Advocacy Instrument) in terms of validity and reliability.

**Methods:**

A multi-step approach was used: Initial translation; Back-translation; Review and synthesis of these translations; *Expert panel* (*N* = 7) rated the prefinal version of the instrument for content validity index (CVI); *International panel* made adjustments guided by the *expert panel*. Reliability testing with test and retest of the adjusted 46-item version was conducted using intraclass correlation coefficient (ICC), weighted kappa (*ҡ*_*Weight*_), sign test, and Cronbach’s alpha coefficient (α), (*N* = 50); and finally Delphi technique procedure with a preselected *Delphi panel* (*N* = 15).

**Results:**

The CVI was determined to be greater than the 0.05 significance level. Item level (I-CVI) ranged 0.82–1.0, with a mean of 0.97. Scale level (S-CVI) on the entire instrument was 0.97. Test-retest procedure was performed to estimate stability. In total, 34 of the items had good-to-high ICC. Accepting an ICC of ≥ 0.70 resulted in a total of 24 items. Homogeneity reliability was estimated by α and was calculated for these items where α = 0.90. In total, 20 of the items had a substantial or almost perfect *ҡ*_*Weight*_ and 23 showed a moderate *ҡ*_*Weight*_. None of the items showed systematical differences. The Delphi technique procedure was used on the 22 items with ICC < 0.70 resulted in adjustments establishing that consensus was achieved.

**Conclusions:**

Undertaking this multi-step, cross-cultural adaptation procedure has effectively ensured that the 46-item Flodén ATODAI [North American version] produces valid and reliable measurements.

## Background

Organ donor advocacy (ODA) attitudes among intensive care unit (ICU) nurses are crucial when championing and respecting the donor’s and donor’s family’s end-of-life decision to donate. ICU-nurses’ awareness, knowledge, skill and competence, i.e. role has an impact upon the organ donation and by that the organ transplantation process. The care by specialist nurses is the key to successful donor management in the critical-care (CC) setting since their actions and behavior are significantly associated with authorization to, or decline of, organ donation (OD) [1–12]. In addition, ICU nurses’ attitudes have an impact on the availability of organs for individuals who need life-saving organ transplant treatment [5, 8, 9].

No studies measuring attitudes toward organ donor advocacy (ATODA) in a clinical context existed before 2011. One reason for this is the absence of validated measuring instruments. In 2011, with the intent to gain an understanding of nurses’ behavior, and their level of ability to advocate for their patients who are either potential or actual organ donors, Flodén et al. [13] developed the 51-item Swedish instrument “Attitudes Toward Organ Donor Advocacy Scale” (ATODAS) to measure ATODA among ICU and CC nurses. This instrument measures ATODA by describing nurses’ actions while caring for potential organ donors and throughout the donation procedure and evaluates changes in organizational structure, guidelines, and educational interventions. The ATODAS is validated in the Swedish context by its application on more than 1200 ICU nurses, i.e. ≥ 50% of all ICU nurses in Sweden. This ATODAS instrument is limited to the Swedish context since it only exists in the Swedish language. Today the ATODAS is to our knowledge the only established instrument within the context of measuring organ donor advocacy, and there is a need for a universal translation among different cultures and countries. Currently, the Flodén ATODAI [North American version] is in use in several countries and continents, and the process of developing a Spanish version has started.

Since specific behavior by ICU personnel is significantly associated with the frequency of referral and OD consent, it is of crucial importance to understand the reasons behind ODA. The concept of ODA in the situation of OD is defined by Flodén et al. [5] as respecting the potential or actual organ donor’s rights, representing, or speaking up for his/her wishes, as well as the family’s points of view, in the OD decision-making process. According to the International Council of Nurses’ Code of Ethics, a nurse’s primary professional responsibility is to people requiring nursing care. Thus, nurses’ behavior and their level of ability to advocate for their patients’ desires applies to potential and actual organ donors [14]. In regard to nurses’ professional ethics in situations when the possibility of OD arises, nurses should represent and defend their patients’ wishes regarding ODA [15]. The relative rarity of OD in any hospital or country makes it important to reach out to an international clinical context to establish developmental changes, i.e. professional and/or organizational. After reviewing the roles and practices of ICU and CC nurses in North America, it became clear that the Swedish ATODAS needed to be adjusted to be used in North American context [16]. Therefore, the aim of this study was to translate, adapt, and establish the psychometric properties of the Swedish ATODAS to one which would be equally valid and reliable in North America. As part of the instrument development in this study, the name of the instrument changed from ATODAS, the 51-item Swedish scale, to Flodén ATODAI (Attitudes Toward Organ Donor Advocacy Instrument), the North American English version.

## Methods

### Design

The study used a methodological design comprising of a cross-cultural adaptation procedure to effectively translate the 51-item Swedish ATODAS instrument for use in other cultural and language settings. Specifically, the study considered the Brislin multi-step approach as best practice [17]. An additional Delphi technique procedure was performed on the Flodén ATODAI [North American version] as a complementary adaptation approach to secure higher scientific certainty of the instrument with regard to validity and reliability by testing the items for content relevance, clarity, and domain coverage [18].

#### Description of the ATODAS [Swedish version]

Flodén et al. [13] developed the Swedish 51-item ATODAS as a means of psychometric evaluation of measuring ICU nurses’ ATODA, including validation and reliability testing. In addition to the demographic data, the instrument contains three dimensions covering statements about attitudes toward: actions to safeguard the wishes of the potential organ donor; actions for supporting the family of the potential organ donor; and actions that promote OD at an organizational or structural level.

#### Translation and cross-cultural adaptation of the Flodén ATODAI [North American version]

The procedure to transfer the Swedish ATODAS instrument into an international arena was guided by Brislin’s [17] multi-step back-translation approach, complemented by a Delphi technique procedure [18] (Table 1).

**Table 1:** The cross-cultural adaptation procedure in six steps according to Brislin [17] and Delphi technique procedure [18] for translating the 51-item Flodén ATODAS [Swedish version] to the 46-item Flodén ATODAI [North American version]

**Step 1:** Initial translation by an English-speaking interpreter.	
**Step 2:** Back-translation by a Swedish-speaking interpreter.	
**Step 3:** Review and synthesis of these translations by an *International committe* of experts.	
**Step 4:***Expert panel* of seven designated ICU nurses rating the instrument; Followed by data analysis I (I-CVI and S-CVI).	
**Step 5:** Test and retest of the prefinal version with 2 weeks in between; Followed by data analysis II (ICC, *ҡ*_*Weight*_, sign test, and Cronbach’s alpha coefficient). In total, 50 ICU nurses from two hospitals in the greater Los Angeles area participated in the test and retest.	
**Step 6:** A preselected panel (*N* = 15) performed an additional Delphi technique procedure for items that showed an ICC < 0.70 in step five. The researchers also made adjustments guided by the panel’s feedback.	

*The first step* was for a professional and native American English-speaking interpreter and bilingual Swedish translator to translate the Swedish ATODAS instrument into American-English. *The second step* comprised of back-translation into American-English, as performed by another expert—a native Swedish-speaking bilingual translator. The translation was performed blindly, i.e. without access to the original version of the Swedish ATODAS.

*Step three* constituted cross-language testing by the *International committe,* consisting of three OD specialists; one representing Sweden (PI), and two representing the United States of America (OneLegacy).

“Review and synthesis of the translations” was performed to provide consensus regarding the most accurate and easily understood items. Working from the original instrument, as well as the translated versions, a synthesis of these translations was conducted to produce a consolidated instrument. This validity-checking procedure ensured the translated version reflected the same item content as the original. A written report thoroughly documented the synthesis procedure by addressing each of the issues and how they were resolved. The consensus included the translated version of the instrument and the introduction and instruction to the instrument, resulting in the prefinal version of the Flodén ATODAI [North American version]. The described procedure included achieving equivalence between the original version and the translated version.

### Study populations: steps four to six

#### Step four

Seven designated ICU or CC nurses in the greater Los Angeles with experiential knowledge of caring for at least one organ donor formed an *expert panel*. The panel evaluated the content validity of the items, with reference to Lynn’s criteria [19]. All seven nurses on the panel were female, aged between 29 and 55 years with a mean age of 44.2 years, and their work experience in the ICU and/or Emergency Department ranged between 5 and 31 years. The panel represented nurses from trauma, education, and teaching hospitals: Three worked in the ICU; three in the Emergency Department; and one in the Education Department. Three of the experts were managers/charge nurses, one was a clinical nurse specialist, one a nurse educator, and two were bedside nurses.

#### Step five

In total, 50 ICU nurses from two hospitals in the greater Los Angeles area participated in the test and retest; one university-affiliated hospital (with different types of ICUs); and one county or community hospital (one ICU) (Table 2). The inclusion criteria were: Being an ICU or CC nurse; experiential knowledge of caring for at least one organ donor; and currently working in a clinical setting with OD. The exclusion criteria were: Being a nurse who was not currently working and/or being a nurse without experience of caring for organ donors.

**Table 2:** Socio-demographics of ICU nurses performing the test-retest of the 46-item Flodén ATODAI [North American version]

ICU or CC nurses	*N* = 50
Age	25–63 years (mean 38 years)
Gender:	
Men	*n* = 13
Women	*n* = 37
Work experiences	0.1–34 years (mean 10.2 years)
Experience of caring for brain-dead patients:	
≤ 5 times	*n* = 13
6–10 times	*n* = 6
≥ 10 times	*n* = 31

#### Step six

A preselected panel of 15 nurses in the United States of America, with extensive experiential knowledge of caring for organ donors, comprised the *Delphi panel* for the purpose of completing the additional Delphi technique procedure (Table 3).

**Table 3:** Socio-demographics of panel (*N* = 15) performing the Delphi technique procedure of the 46-item Flodén ATODAI [North American version]

Location:	
Greater Los Angeles	*n* = 11
Western and South United States	*n* = 4
Age	23–60 years (mean 46.7 years)
Gender:	
Female	*n* = 12
Male	n = 3
Ethnicity:	
Asian	*n* = 7
Caucasian	*n* = 6
Afro-American	*n* = 2
Current workplace:	
Intensive Care (general)	*n* = 8
Emergency Department	*n* = 4
Trauma ICU	*n* = 1
Cardiac ICU	*n* = 1
Neuro ICU	*n* = 1
Main position:	
Bedside nurse	*n* = 14
Charge nurse	*n* = 1
Work experience in ICU	3–32 years (mean 16 years)
Hospital:	
Community hospital	*n* = 12
University hospital	*n* = 2
Trauma hospital	*n* = 1
Private hospital	*n* = 1

### Data collections and analysis: steps four to six

#### Step four: first data collection

The first data collection required testing the prefinal version of the Flodén ATODAI [North American version]. The *expert panel* was given a rating form with the theoretical definition and a delineation of the three dimensions, objectives, and items. They were asked to review the prefinal 51-item version of the Flodén ATODAI [North American version] for content relevance, clarity, and domain coverage and to rate each item on a 4-point scale (from 1 = *not relevant* to 4 = *very relevant*) [19, 20].

#### Step four: first data analysis

##### Content validity

The *expert panel* was formed to estimate the content validity (with reference to Lynn’s criteria [19] of the items. Content was considered valid when an item was rated as either 3 (relevant and needs little revision) or 4 (very relevant) by at least six evaluators (> 86%) and, thus, was included in the new scale [19].

The *International committe* analyzed the content validity rating by the *expert panel* and weighted the scores, which resulted with the prefinal 51-item instrument being reduced to 46 items. Of the remaining 46 items, five items were re-worded, as guided by the recommendations of the *expert panel*. A content validity index (CVI) was calculated to indicate the extent of expert agreement, both for the item CVI (I-CVI) and for the scale CVI (S-CVI). An I-CVI was determined by the number of experts who rated an item content as valid (giving it a rating of 3 or 4) divided by the total number of experts, resulting in a proportion of agreement for each item. The S-CVI was determined by the averages of the I-CVIs [19, 20].

#### Step five: second data collection

The study performed a test-retest procedure to estimate stability (reliability testing) of the adjusted version of the prefinal 46-item version of the Flodén ATODAI [North American version], as developed from the data analysis performed in step four. Fifty ICU nurses agreed to participate by answering the instrument on two occasions, with 2 weeks in between.

#### Step five: second data analysis

##### Test-retest reliability

The intraclass correlation coefficient (ICC) was used to measure the strength of agreement between the test and retest, using ordered categorial data [21]. The level of agreement was confirmed via the weighted form of kappa coefficients (*ҡ*_*Weight*_) [22].

Moreover, the sign test tested for whether systematical differences occur in either direction, described by exact agreement. The test was two-sided and conducted at the 0.05 significance level. The ICC, the *ҡ*_*Weight*_, and the sign test analyses were performed using SAS software version 9.4 (SAS Institute Inc., Cary, NC, USA).

##### Homogeneity and stability reliability

Homogeneity reliability was estimated using Cronbach’s alpha coefficient (α) via SPSS 18.0. According to the conventional rule by Nunnally, this coefficient should at least exceed 0.70 [23].

#### Step six: third data collection

An additional Delphi technique procedure of the Flodén ATODAI [North American version] was performed to test the items for content relevance, clarity, and domain coverage. The Delphi technique is evidently dependent on the experiential knowledge of its *expert panel* (i.e. the Delphi panel). In this step, the *Delphi panel* only reviewed and judged the 22 items which were identified in step five as having an ICC ≤ 0.70. The preselected *Delphi panel* (*N* = 15) was used to further improve the feasibility of the instrument [18]. The *Delphi panel* members were asked to individually review and judge these 22 items in the Flodén ATODAI [North American version] in two occasions, referred to as “round I” and “round II”. The nurses rated each item using a 4-point rating scale (1 = not relevant; 2 = unable to estimate relevance without item revision or item in need of such revision that it would no longer be relevant; 3 = relevant but needs minor alteration; 4 = very relevant and succinct) [19]. In round II, the *Delphi panel* members reviewed the adjusted version of the Flodén ATODAI [North American version] after round I.

#### Step six: third data analysis

After round I, this study’s primary investigator (PI) summarized and analyzed all participants’ recommendations. The *Delphi panel* rated all 22 items as either “relevant but needs minor alteration [3]”, or “very relevant and succinct [4]”. Four of the items were recommended to be kept as they were. The PI, in consultation with the co-investigators, adjusted and re-worded the remaining 18 items, guided by the *Delphi panel* members’ recommendations. After round II, the PI, in consultation with the co-investigators, summarized, analyzed, and adjusted the Flodén ATODAI [North American version], again guided by the recommendations of the panel.

## Results

### Content validity

The first data collection comprised of the *expert panel* rating the prefinal version of the Flodén ATODAI [North American version]. The *International committe* then analyzed this data for CVI by calculating both the I-CVI and the S-CVI. The I-CVI ranged from 0.82 to 1.0, with a mean of 0.97, while the S-CVI averaged 0.97. This meant the CVI was ≥ 0.78 and, therefore, content validity was established beyond the 0.05 significance level, which is the required criterion according to Lynn [19] and Polit and Beck [20]. This resulted in a reduction of the number of instrument items from 51 to 46. Of the remaining 46 items, five were re-worded using the recommendations of the *expert panel.*

### Test-retest reliability

The strength of agreement between the test and retest was calculated both by ICC and by *ҡ*_*Weight*_. Also, the sign test was used to identify whether any systematical differences had occurred. The 46-item Flodén ATODAI [North American version] showed an ICC between 0.268–0.911 (Table 4). In total, 34 of the items had a good or excellent ICC: Good *n* = 18 (0.60–0.74); Excellent *n* = 16 (≥ 0.75). Accepting an ICC ≥ 0.70 [24] yielded a total of 24 items. The level of agreement was confirmed by *ҡ*_*Weight*_, varying between 0.25–0.87 (Table 5). In total, 20 of the items had a substantial or almost perfect *ҡ*_*Weight*_: Substantial *n* = 17 (0.61–0.80); Almost Perfect *n* = 3 (0.81–0.99). Moderate *ҡ*_*Weight*_ (0.41–0.60) was shown for 23 items. Only three items (2, 4, and 37) showed a *ҡ*_*Weight*_ with fair agreement (0.21–0.40). However, these three items had a high exact agreement that ranged between 75.5–84.0%. Exact agreement between test and retest for all items varied between 52.1–97.9%. None of the items showed statistically significant systematic changes. The retest values were systematically higher for most of the items. The ICC values were, as is to be expected, very similar to those of *ҡ*_*Weight*_ [25].

**Table 4:** The strength of agreement between the test and retest of the 46-item Flodén ATODAI [North American version], as measured by ICC using ordered categorial data [21]

Excellent correlation (0.75–1.00)	*n* = 16
Good correlation (0.60–0.74)	*n* = 18
Fair correlation (0.40–0.59)	*n* = 9
Poor correlation (< 0.40)	*n* = 3

**Table 5:** The level of agreement between the test and retest of the 46-item Flodén ATODAI [North American version] by *ҡ*_*Weight*_ [22]

Almost perfect agreement (0.81–0.99)	*n* = 3
Substantial agreement (0.61–0.80)	*n* = 17
Moderate agreement (0.41–0.60)	*n* = 23
Fair agreement (0.21–0.40)	*n* = 3
Slight agreement (0.01–0.20)	–
Less than chance agreement (< 0)	–

### Homogeneity and stability reliability

Homogeneity reliability was estimated using α for the 24 items identified with an ICC ≥ 0.70 was α =0.90. None of the items had a greater α coefficient “if item was deleted”, meaning that none of the items would substantially affect reliability if they were removed. Furthermore, all items had a “corrected item-total correlation” of 0.30 or above. Calculating the Cronbach’s alpha of the retest gave an α coefficient of 0.913. Analysis of the results of the test and retest established a reasonable degree of both stability and homogeneity for the 24 items in the Flodén ATODAI [North American version] with an ICC ≥ 0.70 for test-retest reliability. Out of the remaining 22 items, 10 had an ICC ≥ 0.60 (i.e. good correlation), 9 had a fair ICC (0.40–0.59), while 3 had a poor ICC (< 0.40). All items had been rated as “relevant and succinct” or “relevant but needed minor alteration” by the *expert panel*. The majority of the 22 items with ICC < 0.70 needed minor alterations as part of the Swedish to North American cross-cultural adaptation. These alterations were performed using a Delphi technique procedure.

### The Delphi procedure

The Delphi procedure focused on the 22 items identified in step five as showing an ICC < 0.70. During round I*,* the *Delphi panel* rated all 22 items as relevant, recommending 16 for minor alterations. Then, during round II, the *Delphi panel* members reviewed the adjusted items from round I and recommended further changes. The adjustments after both round I and round II involved emphasizing the core of the items via re-wording. The PI performed the final adjustments of the items, guided by feedback from the *Delphi panel*, until consensus was achieved.

## Discussion

Before 2011 no studies measuring ATODA existed in the worldwide clinical context, which is why the Swedish 51-item ATODAS was developed and validated by Flodén et al. [13] and became the first instrument to measure ATODA among ICU and CC nurses. For a worldwide use the primary limitation of the ATODAS is linguistic since it is written in Swedish, but socio-cultural and legal limitations also exist. To address these limitations and allow the scale to be used in an international clinical context, a systematic translation procedure for cross-cultural adaptation was initiated and performed [17, 26] (Table 1).

The term “cross-cultural adaptation” is used to encompass a procedure that takes into consideration issues related to both language (translation) and cultural adaptation during the process of preparing an instrument for use in another setting [26]. To merely translate an instrument word for word into a different language does not adequately take into account the cultural and linguistic differences. The content of the items needs to be relevant in each culture, for example legislations and government regulations. The “cross-cultural adaptation” term consist of three parts: 1st, the preparation of the instrument for use in another culture, language and country; 2nd, the use of target populations to evaluate clarity in the instructions, response format and the items of the instrument; and 3rd, the field testing of the pre-final version of the instrument [17, 26–30]**.**

### The process of preparing the Flodén ATODAI for use in the North American context

Application of an instrument in a different culture, language and country would necessitate translation and cultural adaptation [17, 26–30]. The procedure used for the linguistic translation included two different professional interpreters for the translation and back-translation. As a validity check and to ensure a high-quality translated instrument, the *International committe* of experts safeguarded that the translated version reflected the same item content as the original version and that the content was relevant in the context of North America. In situations where there were uncertainties around the meaning of specific words or items, a strength was that the developer of the original instrument was a member of the *International committe* and could provide clarifications when needed [26, 31]. Items in the original Swedish 51-item ATODAS that were related to Swedish legislation and how they shall be applied in the Swedish context were removed. For example, Sweden has a presumed consent to organ donation, and therefore items were removed that asked about approaches and actions to respect the legislated presumed consent and how to balance this legislation toward the wishes of the family. Moreover, items covering organizational structure were adjusted to fit the North American context. In Sweden, when a potential organ donor is identified, the ICUs call the Transplant coordinator on call, which is an in-hospital position linked to the transplant institutes. By contrast in the United States of America, the ICUs call their organ procurement organization (OPO). In Sweden it is considered ethically insensitive to ask about culture, religion, and country of origin. However, the first issue raised by the American members of the *International committe* was the lack of these topics. Therefore, the descriptive items were adjusted in the cross-cultural adaptation by adding the items of culture, religion, and country of origin to the North American version of the Flodén ATODAI. The literature supports the procedure chosen in this study to ensure that the items were translated correctly and were relevant in the new setting [17, 26–30, 32].

### The use of target populations

To return to the target populations and have experts in the field discuss the nuances brought out by the different versions of the translated instrument throughout the process is crucial, and the instrument should be adjusted accordingly after a consensus is reached [26, 30, 33, 34]. In our study an *expert panel* was used to evaluate the content validity following the recommendations by Lynn on how to quantify an otherwise subjective process [19]. It showed that we achieved strong content validity that was culturally relevant. The use of the Delphi panel was an additional approach to return to the target population. The use of the Delphi technique procedure is not included in the original approach by Brislin [17] but was added to this study to increase the test-retest reliability of the Flodén ATODAI [North American version]. The main advantage of the Delphi technique procedure is the capability of guiding the Delphi panel opinion toward achieving consensus in a given area of uncertainty or lack of empirical evidence [18, 33, 35]. The panel size, the composition of panel members as well as the qualification of the experts are important factors to make a valid contribution. To secure higher scientific certainty of the instrument, we followed the recommendations described by Powel by having a heterogeneous group of panel members (Table 3) and followed the recommended procedure consisting of two subsequent rounds [18].

### Field testing of the pre-final Flodén ATODAI [North American version]

Field testing and refining the instrument with members of the target populations is needed to ensure that items are translated correctly and are relevant in the new setting [26–30]. Test-retest was used to measure the strength of agreement between the test and retest [21–23]. The results concluded that reliability was established due to the test-retest reliability testing, where 34 of the items had a good or excellent ICC and the *ҡ*_*Weight*_ confirmed the level of agreement. Only three items showed a *ҡ*_*Weight*_ with fair agreement, but all these three items had a high exact agreement between the test and retest. Moreover, the Cronbach’s alpha coefficients showed excellent homogeneity and stability reliability.

In a situation where the possibility of OD occurs in the ICU, the nurses are expected to enable the donation, within the boundaries of professional ethics [14, 15]. The key to successful donor management in the CC setting is significantly associated with the ICU and CC nurses’ attitudes toward OD, having an impact on the availability of organs for individuals who need life-saving organ transplant treatment [1–12]. The intent of creating the Flodén ATODAI [North American version] was to develop an instrument capable of supporting ICU and CC nurses in gathering knowledge and to develop best practices and guidelines when advising and assisting (potential) organ donors and their families, i.e. support attitudes and actions toward ODA.

After review of the role and practice of the ICU and CC nurses in the United States of America, it became obvious the Swedish ATODAS needed adjustment before it could be used in North America, to fit its socio-cultural and legal context. The main differences between the Swedish version and the North American version of the instrument are due to national context, e.g. legislation and guidelines. The methodological procedure performed, revealed evidence of validity and reliability for the Flodén ATODAI [North American version]. Thus, this multi-step approach has effectively ensured a stronger scale for assessing attitudes toward ODA (the Flodén ATODAI [North American version]).

Clinical benefits of the Flodén ATODAI [North American version] enable identification of educational and organizational needs and may be useful to evaluate organizational changes, and if such changes will be sustainable over time. Therefore, the instrument assists the professional development of ICU nurses. The Flodén ATODAI [North American version] opens the possibilities to use this instrument to perform studies within the North American context, as well as globally to measure ATODA.

## Conclusions

The translated and tested instrument Flodén ATODAI [North American version] was adapted to be culturally relevant, yielding valid and reliable results for use in a clinical North American context within a global perspective. Undertaking this multi-step approach has effectively ensured that the cross-cultural adaptation procedure resulted in a stronger instrument for valid and reliable measurements. The North American version of the Flodén ATODAI provides a framework for researchers in general, but clinicians in particular, choosing to utilize this instrument for work in other cultural and geographic settings. Study limitations are that content validity of the 46-item Flodén ATODAI needs to be further scrutinized. Therefore, the next step should be to use the instrument in a large-scale study within the United States of America and implement factor analysis to determine construct validity.

## Data Availability

The datasets used and/or analyses during the current study are available from the corresponding author on reasonable request.

## Abbreviations

ATODA: Attitudes Toward Organ Donor Advocacy
ATODAI: Attitudes Toward Organ Donor Advocacy Instrument
ATODAS: Attitudes Toward Organ Donor Advocacy Scale (in Swedish)
CC: Critical Care
ICC: Intraclass correlation coefficient
ICU: Intensive Care Unit
I-CVI: Item - Content Validity Index
OD: Organ Donation
ODA: Organ Donor Advocacy
PI: Primary Investigator
S-CVI: Scale - Content Validity Index
